# Role of Optical Coherence Tomography Angiography to differentiate Intraretinal microvascular abnormalities and retinal neovascularization in Diabetic Retinopathy

**DOI:** 10.12669/pjms.38.1.3891

**Published:** 2022

**Authors:** A. Sami Memon, Nasir A. Memon, Pir Salim Mahar

**Affiliations:** 1 Dr. Abdul Sami Memon, FCPS. Assistant Professor, Aga Khan University Hospital, Karachi, Pakistan. Isra Postgraduate Institute of Ophthalmology, Karachi, Pakistan; 2 Dr. Nasir Ahmed Memon, FCPS. Assistant Professor, Isra Postgraduate Institute of Ophthalmology, Karachi, Pakistan; 3 Prof. Dr. P.S. Mahar, FRCS, FRCOphth. Professor of Ophthalmology, Aga Khan University Hospital, Karachi, Pakistan. Isra Postgraduate Institute of Ophthalmology, Karachi, Pakistan

**Keywords:** Intra retinal microvascular abnormalities, Optical coherence tomography angiography, Proliferative diabetic retinopathy, Swept source Optical coherence tomography

## Abstract

**Objective:**

To assess proliferative diabetic retinopathy (PDR) and to describe the difference in angiographic representation of new vessels (NVs) and Intra retinal microvascular abnormalities (IRMA) on optical coherence tomography angiography (OCTA).

**Methods:**

A cross-sectional observational study was performed at ISRA Postgraduate Institute of Ophthalmology, Karachi, from March 2018 to September 2018. Forty-two eyes of 21 patients with history of diabetes mellitus (DM) were examined. Twenty-eight eyes with a clinical diagnosis of severe non proliferative diabetic retinopathy (NPDR) or proliferative diabetic retinopathy (PDR) according to early treatment diabetic retinopathy study (ETDRS) were included and evaluated using Swept source optical coherence tomography angiography (SS-OCTA). Then face wide field SS-OCTA images and co registered structural optical coherence tomography (OCT) with flow overlay were used to distinguish the features of IRMA and retinal NVs.

**Results:**

Forty-two eyes (21 patients) were examined clinically. Fourteen eyes had moderate NPDR, 15 had severe NPDR and 13 eyes had changes consistent with PDR. After clinical diagnosis, we included 28 eyes in our study based on inclusion criteria. These 28 eyes went through SS-OCTA evaluation and we observed 15 cases with PDR and 13 with severe NPDR changes. The OCTA and clinical diagnosis were similar except in 2 eyes, which is critical but not statically significant showing the importance of this noninvasive technology.

**Conclusions:**

Widefield OCTA can work as an alternative to fundus fluorescein angiography (FFA) in the diagnosis of diabetic retinopathy (DR). As it is a non-invasive and depth encoded technique so can be used frequently to monitor the retinal changes and their progression.

## INTRODUCTION

Diabetic retinopathy (DR) is one of the leading complications of diabetes mellitus (DM) with the treatment philosophy based on the severity of the retinal changes. There are approximately 93 million people with DR worldwide, out of which 17 million have proliferative diabetic retinopathy (PDR).^1^ The National Survey of blindness conducted in Pakistan in 2007 estimated that there were at least 90,000 to 100,000 adults with vision threatening diabetic retinopathy (VTDR) requiring eye care.^2^ The prevalence of DR is expected to increase due to the use of more sensitive detection tools such as optical coherence tomography (OCT), optical coherence tomography angiography (OCTA), ultra-wide fundus photography and angiography. DR is classified into non proliferative and proliferative stages. The non-proliferative diabetic retinopathy (NPDR) manifests microvascular changes, including micro aneurysms, intra retinal hemorrhages, venous beading and intra retinal microvascular abnormalities (IRMA), whereas proliferative diabetic retinopathy (PDR) is identified by retinal neovascularization (NV) that develops in response to retinal ischemia. NVs are bunch of fine vessels lying on the surface of the retina, while Intra retinal microvascular abnormalities (IRMA) are defined as collateral vessels arising from the preexisting capillaries and are located entirely within the retina and are difficult to distinguish from early extra retinal neovascularization. Early detection of severe NPDR and early PDR is very important in DR treatment as the chance of progression to vision threatening diabetic retinopathy (VTDR) within one year is calculated at 50 percent.^3^ Optical coherence tomography (OCT) is a non-invasive technique and has gained popularity over the last two decades for acquiring cross sectional retinal images. It is based on the principle of optical reflectometry, which involves the measurement of light back-scattering through transparent or semi-transparent media such as retina.^4^ It is useful for the diagnosis and monitoring of DR, especially diabetic macular edema (DME). However, we cannot visualize new vessels with structural OCT scans.^5^,^6^ To overcome this limitation, OCTA has gained popularity to map out retinal and choroidal vasculature. It is a non-invasive, rapid, depth encoded technique and is without the use of fluorescein dye. Then face OCTA can easily visualize the vascular structure of retina and choroid and is ideal noninvasive tool for diagnosis, management and monitoring of different stages of DR, Age related macular degeneration (ARMD) and other chorioretinal vascular disorders.^7^,^8^

It is important to diagnose and distinguish severe NPDR and PDR and treat it early to prevent vision loss from nonvascular complications so en face OCTA with structural OCT is a useful technique to distinguish between NVs and IRMAs. The aim of this study was to differentiate NVs and IRMAs with this noninvasive technique in patients with diabetic retinopathy.

## METHODS

This cross-sectional observational study was performed at ISRA Postgraduate Institute of Ophthalmology, Karachi, from March 2018 to September 2018. Forty-two eyes of 21 patients with history of DM were examined. Twenty-eight eyes with a clinical diagnosis of severe NPDR or PDR according to early treatment diabetic retinopathy study (ETDRS) were included.^9^ The study approval was granted by Institute’s Ethical Committee on 18^th^ January 2018 with protocol number A-00044/A and was carried out according to the declaration of Helsinki.

All patients provided informed consent to participate in this study and underwent comprehensive ophthalmologic examination, including measurement of the best-corrected visual acuity (BCVA), intraocular pressure (IOP), slit-lamp bio microscopy and fundus examination with 90 diopter lens. All included cases of PDR and severe NPDR were evaluated with SS OCTA (Topcon DRI OCT Triton, Japan). This device has an image acquisition speed of 100,000 A-scans per second, an axial resolution of 8µm, and transverse resolution of 20 µm. The swept-source OCT has a longer wavelength at 1,050 nm with less signal roll off at increased imaging depth compared with spectral domain OCT20-26. This device has both conventional OCT, en face OCT and OCTA capability. The enface widefield SS-OCTA images and the co registered structural OCT with flow overlay were used to distinguish the features of IRMAs and retinal NVs. The term flow overlay means flow on the cross-sectional OCT images5.The 12/12 mm SS-OCTA scan pattern data were used to find difference between IRMA and NVs. Patients with any other retinal disorders, including history of vitreoretinal surgery, glaucoma, uveitis and presence of media opacities, such as severe vitreous hemorrhage, cataract and corneal opacity and also with previous history of Intravitreal injection of anti-VEGF, steroids and laser were excluded.

### Statistical analysis:

Statistical analysis was done using SPSS version 23.0. All continuous variables were presented as mean with standard deviation. The Categorical data is presented as frequency and percentages. Chi square test was applied to see the significance of OCTA for grading of DR and qualitative features of IRMAs and NVs. A p-value < 0.05 was considered to be statistically significant.

## RESULTS

**Fig.1:**
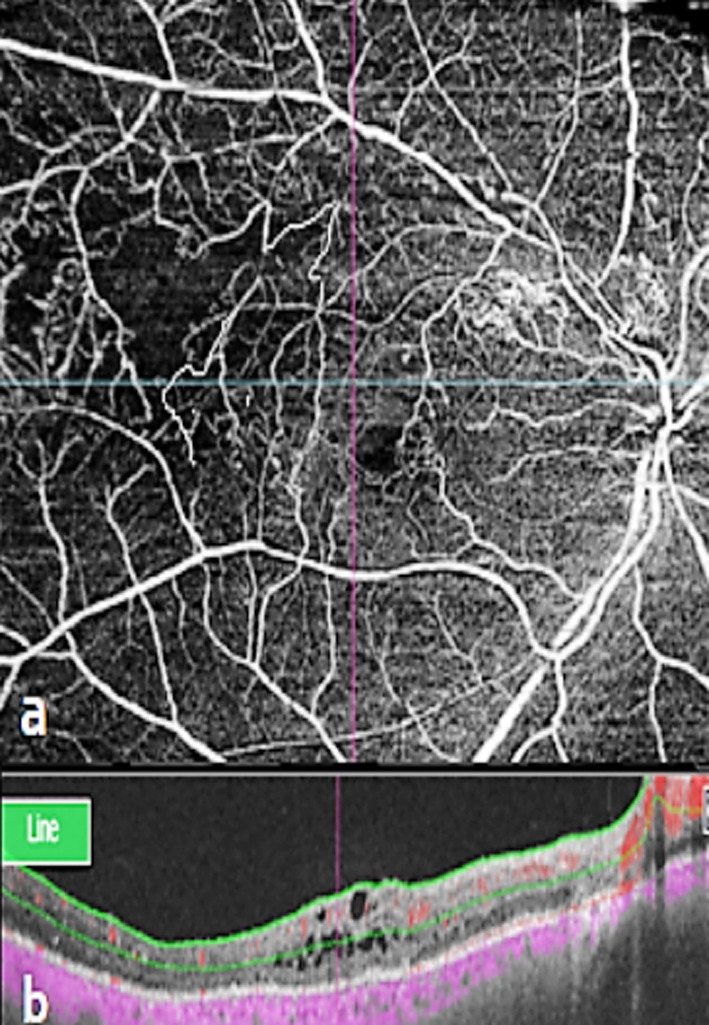
Intra Retinal Microvascular Abnormalities. **a.** Magnified 12mm×12mm en face OCTA showed IRMA, **b.** Co-registered OCT with flow overlay of OCTA data confirms intra retinal flow below the ILM.

**Fig.2:**
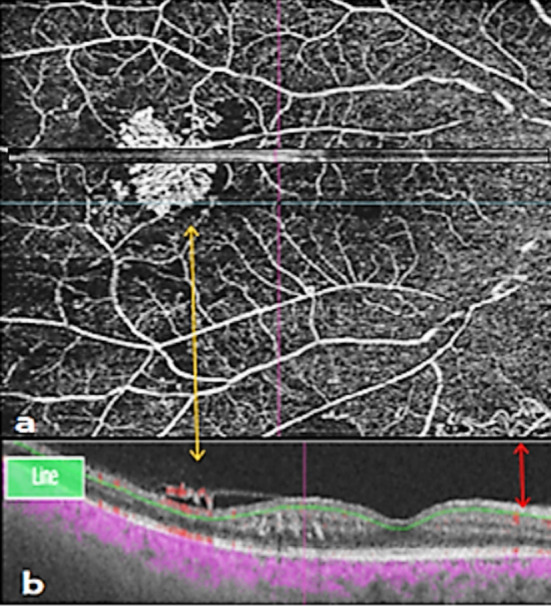
Retinal new vessels and Intra retinal microvascular abnormalities. **a**. Magnified en face 12 mm ×12 mm OCTA of two retinal lesions, yellow arrow showing new vessels (NV) and Red arrow representing Intraretinal microvascular abnormalities (IRMAs), **b.** Flow overlay of OCTA confirmed suprarenal and intraretinal flow.

**Fig 3:**
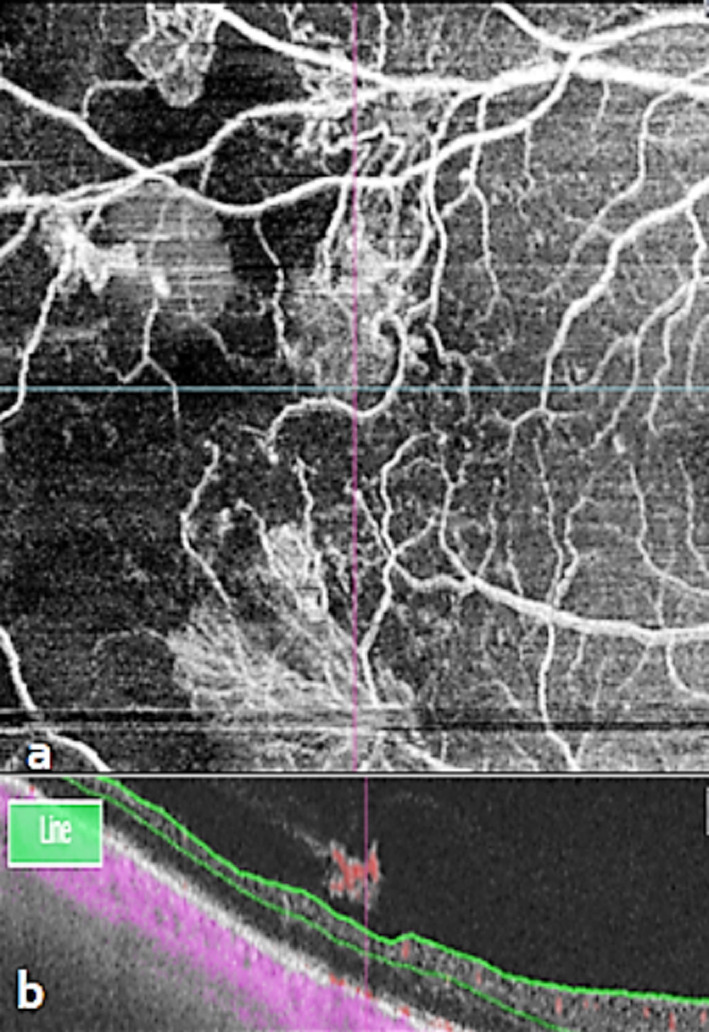
New Vessels adjacent to areas of capillary loss. **a**. Magnified 12mm×12mm en face OCTA showed capillary loss, **b**. Co-registered OCT with flow overlay of OCTA found advance NVs.

Twenty-one patients were included in this study. Out of which, 11 (52%) were male and 10 (48%) were female. Mean age of the patients was 50.71 ± 9.249 years with range of 37-65 years. Overall, 42 eyes (21 patients) were examined clinically, with 14 eyes having moderate NPDR, 15 had severe NPDR and 13 eyes with changes consistent with PDR. After clinical diagnosis, we included 28 eyes in our study based on inclusion criteria. These 28 eyes went through SS-OCTA evaluation with 15 eyes showing PDR and 13 eyes with severe NPDR changes. The OCTA and clinical diagnosis were almost similar except in 2 eyes, which is critical with P-value 0.001 (Table-I). We observed 56 irregular abnormal looking vasculatures on enface OCTA and co registered OCT with flow overlay of OCTA. Out of these 56 lesions, 36 (64%) were IRMAs (Fig.1 and Table-II) found restrained under the ILM with intra retinal flow. Twenty lesions (36%) were NVs (Fig.2), Out of 20 lesions, 18 (90%) were in advanced stage penetrating ILM and posterior hyaloid (Fig.3) and 2 (2%) lesions were found only breaching ILM.

**Table I:** Clinical and OCTA diagnosis.

Clinical Diagnosis	OCTA Diagnosis		Total	P-value

	PDR	Severe NPDR		
PDR	13	0	13	0.001
	46.4%	0.0%	46.4%	
Severe NPDR	2	13	15	
	7.1%	46.4%	53.6%	
Total	15	13	28	
	53.6%	46.4%	100.0%	

*Chi-square test was applied to see the significance. OCTA: Optical coherence tomography angiography, PDR: Proliferative diabetic retinopathy, NPDR: Non-proliferative diabetic retinopathy.

**Table II:** OCTA findings and Abnormal Vascular Lesions.

OCT abnormal vascular Lesions	Supra retinal flow		Total	P-value

	NO	YES		
IRMA=1	36	0	36	0.001
	64.3%	0.0%	64.3%	
NV=2	0	20	20	
	0.0%	35.7%	35.7%	
Total	36	20	56	
	64.3%	35.7%	100.0%	

*Chi-square test was applied to see the significance, OCT: Optical coherence tomography, IRMA: Intraretinal microvascular abnormalities, NV: Neovascularization.

## DISCUSSION

The DR remains asymptomatic until the pathology is significantly advanced. It is therefore imperative to detect retinal changes at an early stage.^10^ Sometimes IRMA may be difficult to find clinically, because the appearance could be restrained, peripheral and masked by the presence of retinal hemorrhages. It is important therefore to distinguish between IRMA and retinal NVs to determine diagnosis, prognosis and treatment plan.^10^ In this study we reviewed histopathologic definition of NVEs in DR and demonstrated the use of SS OCT and OCTA for the diagnosis of severity of DR. ETDRS has highlighted the use of fundus fluorescein angiography (FFA) in DR and has also described its importance in the diagnosis of macular edema and capillary Non perfusion.^11^ FFA is important and useful test for assessing NVs, but leakage in FFA alone may not be enough for differentiating IRMAs from NVs, because leakage can also occur in other vascular disorders. Furthermore, FFA is an invasive test, time consuming, costly and has potential for severe adverse effects varying from mild nausea and vomiting to life threatening complications.^12^ In 1997, OCT made revolutionized change in ophthalmology especially in the field of vitreo-retina. With this noninvasive technology we can evaluate the disruption of ILM associated with NVE.^13^-^15^ It still remains debatable that IRMAs are precursors of NVE but it is eminent that the IRMAs increase the risk of PDR. Lee et al.^16^ described the conversion from IRMA to NVE and suggested that once there is a breach in ILM, the early NVs will grow towards posterior hyaloid. In our study we have observed multiple irregular vasculatures on enface OCTA and co registered OCT with flow overlay of OCTA, most of them are IRMAs, because these lesions remain under ILM with intra retinal flow and showing no breach in ILM. Ishibazawa and colleague^17^ described NV on enface OCT angiography, describing two distinct morphologic features of the new vessels. Firstly, most new vessels were irregular proliferation of fine vessels, which were defined as exuberant vascular proliferation (EVP). The second one was tiny vascular loops of vessels. In our study we found 90% fine vessels and most of them penetrating ILM and posterior hyloid and can be explained as EVP. De Carlo et al.^18^ have explained that retinal NVs appear near to retinal non-perfusion areas and Arya and coworkers^3^ has added that NVs develop in response to ischemia and may be originating from IRMA and If these vessels breach the ILM, they are defined as retinal NV with potential to proliferate. If we accept this theory, our study has similarly showed the retinal NVs and IRMAs present next to the areas of capillary loss (Fig.3), making enface OCTA useful in differentiating NPDR from PDR, and helping in treatment planning with monitoring of the disease intensity.^19^ Retinal NVs can be appreciated on enface OCTA via observation of flow signal above the ILM22. En face OCTA can identify early retinal NVs20 and can recognize the origin and morphological pattern of NVs in PDR. OCTA is also able to detect subtle NVs, which are difficult to differentiate from microanerysms on FFA.

Lee et al.^16^ has also described stages of NVs, with stage I defined by the hyper reflective posterior hyaloid layer appearing intact despite the presence of the NVs through the break of the ILM not extending into vitreous cavity, in Stage II, NVs are defined by the growth along the posterior hyaloid. In stage III, NVs appear to involve multiple areas with breach of ILM and growth in different planes. Our study has showed NVs growing along the posterior hyaloid can be considered stage II, according to the Lee’s classification. Pan and colleague^20^ have categorized three subtypes of retinal NVs in PDR, based on OCTA images. Type-I NVEs are tree shaped originating from the venous circulation, Type-II are octopus shaped arising from the capillary network, while Type-III have sea fan shape with origin from IRMA. In our study we didn’t find the actual origin of NVs. Schaal et al.^21^ compared vascular abnormalities in DR with SS-OCTA to color fundus photography and explained variability between these modalities. Our study has established that IRMA and retinal NVs can be differentiated by using Wide field OCTA. Wide field OCTA could be a reasonable alternative to FFA in the assessment of IRMA and NVs, for either DR screening or to monitor high risk DR eyes.

### Limitations of the study:

Limitations of this study include, small number of cases and non-comparison with FFA frames. In future software advancement may improve the utility of wide field OCTA for DR screening making grading of DR easy in automated fashion.

## CONCLUSION

The OCTA can potentially work as an excellent alternative to FFA in the diagnosis of DR because of its non-invasive and depth encoded technique. It can therefore be used frequently to monitor the progression of the disease and distinguish between IRMAs and NVs.
